# Analysis of the use, effectiveness, and efficiency of the pick and roll in elite women’s basketball

**DOI:** 10.3389/fspor.2025.1553270

**Published:** 2025-05-01

**Authors:** M. Amatria, I. Iván-Baragaño, J. L. Losada, R. Maneiro

**Affiliations:** ^1^ Pontificial University of Salamanca, Salamanca, Spain; ^2^ Department of Sport Sciences, Faculty of Medicine, Health, and Sports, Universidad Europea de Madrid, Madrid, Spain; ^3^ Department of Social Psychology and Quantitative Psychology, University of Barcelona, Barcelona, Spain; ^4^ Department of Special Didactics, University of Vigo, Vigo, Spain

**Keywords:** basketball, women, success, multivariate logistic regression, observational methodology

## Abstract

This study investigates the effectiveness and outcomes of “pick and roll” plays in elite women's basketball, focusing on the 2021-2022 EuroLeague Final Four. The main objective is to analyze the tactical efficiency of these actions and identify the most effective resolution strategies. A total of 298 plays from 1,757 events across four matches were examined using an observational methodology, adhering to a Nomothetic, Point, and Multidimensional (N/P/M) design. An observational instrument consisted on twenty-three criteria and 126 categories was developed to codify the actions, with reliability confirmed by a Cohen's Kappa value of 0.93, indicating “almost perfect” agreement. Results show that 71.8% of “pick and roll” plays concluded with a shot, with the highest success rates achieved when the screener executed the final attempt near the basket. Actions with fewer passes post-screen were significantly more effective, and collective strategies, such as passing to the screener, outperformed individual efforts by the ball handler. In addition, the multivariate results highlight the second and third quarters of the game as the most important when it comes to executing an effective offensive pick & roll. These findings emphasize the importance of immediate actions post-screen, tactical fluidity, and proximity to the basket, providing valuable insights to enhance offensive strategies in competitive women's basketball.

## Introduction

The pick and roll in basketball represents one of the most commonly used and effective offensive strategies to gain an advantage over the opposing defense (1). This technical-tactical element involves the intervention of two players from the same team, with different roles, converging at a specific moment in the same space. One of them, the player without the ball—often a center—sets a screen for the teammate handling the ball, complicating the task of the defender guarding the ball handler (2). This play facilitates the creation of defensive mismatches, generates spaces for shots, drives, or passes, and promotes quick decision-making, an essential aspect of modern basketball (3). Furthermore, the pick and roll effectively adapts to different tactical scenarios, serving as a key component in both structured offensive systems and fast transitions (4). Its proper execution requires precise synchronization, dynamic understanding of the game, and effective coordination between the players involved, reaffirming its central role in contemporary basketball (5).

Research on collective tactics in basketball highlights the pick and roll as an essential resource (1, 6). Nunes et al. (7) and Bardavío et al. (8) emphasize its strategic relevance due to its frequency and effectiveness. Nunes et al. (7) documented that over 25% of actions in the ACB league involve a pick and roll, with an effectiveness close to 90%, consolidating it as a crucial factor in team success. Similarly, Gómez et al. (9) identified that the direction of the screen, timing, and spacing are key determinants of its effectiveness, as well as offensive players' ability to adapt to the opposing defensive strategy.

In a related study, Bardavío et al. (8) identified ten distinct patterns linked to the pick and roll, each with a 90% probability of success. Their findings emphasize the significance of enhancing creativity in these plays to optimize advantage recognition and improve players' spatiotemporal coordination. They also noted that perimeter players tend to benefit more from employing the pick and roll, with teammates' off-ball movement being critical to enhancing its effectiveness. Morillo-Baro et al. (10) indicated that the pick and roll is present in 30%–45% of positional attacks in both men's and women's basketball, though its usage drops to 8% in transition situations. Additionally, they found that female players show greater tactical diversity at the start of these actions, demonstrating a wider range of strategic choices and adaptations to different game situations.

Regarding its distribution during games, pick and roll plays occur uniformly across all four quarters (7). During possessions, 61.3% of these plays occur in the middle phase, with continuity via passing in 61.5% of cases, while only 17.7% end in a shot. Moreover, 98% of pick and rolls are executed facing forward, compared to 2% executed backwards, with no significant differences based on the temporal score (47.9% for winning teams and 47.4% for losing teams). Unexpectedly, losing teams perform a greater number of effective pick and rolls than winning teams. Marmarinos et al. (11) support this observation, suggesting that the quality of execution of this play might correlate with final standings.

Despite these findings, there are still aspects that require further exploration regarding this technical-tactical action, such as the number of passes following the screen execution and their influence on the play's outcome, or whether the screener's intervention or a third player's involvement is more effective in finalizing the offensive action in women's basketball.

Although elite women's basketball has garnered academic interest, the volume of research remains significantly lower compared to other sports or men's basketball. Existing studies primarily focus on performance analysis (12), biomechanics (13), psychological factors (14), and training strategies (15), as well as social and gender-related issues (16) and tactical studies (17, 18).

Given the above, generating new knowledge about women's basketball, complemented by identifying tactical elements associated with collective performance, would significantly enhance training and its subsequent application in competition. Therefore, the primary aim of this research was to analyze the effectiveness of direct screens in elite women's basketball and describe the most effective finishing methods. To this end, two types of analysis have been carried out: one bivariate to determine the statistically significant relationships in relation to the dependent variable considered, and secondly, a logistic regression analysis has been carried out to determine the possible existence of a theoretical model.

## Method

This study follows the N/P/M research design framework described by Anguera et al. (19). The design is classified as Nomothetic (as it analyzes multiple teams), Point (as it focuses on a specific competition phase, i.e., the Final Four of the 2021–2022 Women's Basketball EuroLeague) and Multidimensional (as it considers various criteria and categories to capture behavioral patterns). The observation instrument used ensures both exhaustiveness (covering all relevant behaviors) and mutual exclusivity (each behavior fits into only one category).

### Participants

The participants for this study were selected through observational or convenience sampling (20), whereby all pick and roll plays that occurred during the four games comprising the Final Four of the 2021–2022 Women's Basketball EuroLeague championship (two semifinals, the third-place game, and the final) were included. The participating teams were Perfumerías Avenida (11 players), Sopron (11 players), USK Prague (11 players), and Fenerbahçe (12 players), with a total of 45 players participating in all. The total number of multi-events recorded, forming the sample for this study, was 1,757, which resulted in a total of 298 pick and roll plays executed across the four analyzed games. The images of the games were obtained from public television images. Specifically, under subscription to the NBA.com platform, namely the paid subscription *nbaleaguepass.*

This manuscript did not require informed consent or approval from an ethics committee, in accordance with the guidelines established by the National Commission for the Protection of Human Subjects of Biomedical and Behavioral Research (21). The study is limited to the observation of public sequences where participants have no reasonable expectation of privacy and does not involve any interventions by the researcher or direct interaction with the subjects. Furthermore, the fundamental ethical principles for research involving human subjects were upheld in strict compliance with the Declaration of Helsinki (22–24).

### Observational instrument

The instrument, developed *ad hoc*, is a combination of a field format and category systems (25) (Table 1). The requirements established by Anguera et al. (26) were followed for its construction, resulting in an observation instrument comprising 23 criteria and 126 categories.

**Table 1:** Observation instrument.

N°	Criteria	Categories: codes and brief description
1	Phase	SMF1) Semifinal 1; SMF2) Semifinal 2; Consolation) 3rd and 4th Place Game; Final) Final
2	Quarter	FIRSTQ) First Quarter; SECONDQ) Second Quarter; THIRDQ) Third Quarter; FOURTHQ) Fourth Quarter; OVERT) Overtime
3	Time	S24) Final 24 s; S16) Final 16 s; S8) Final 8 s
4	Partial outcome	TIE) Tie; WIN) Winning; LOS) Losing
5	Type of collective defense	MxM) Man-to-man; ZON) Zone; MIX) Mixed; CR) Circumstantial
6	Type of offense	CONT) Counterattack; TRAN) Transition; POS) Positional
7	Players involved	INSOUT) Inside to outside; INSINS) Inside to inside; OUTINS) Outside to inside; OUTOUT) Outside to outside
8	Action before the ball screen	BLOQNBOT) Screen to a player who hasn't dribbled; BLOQBOT) Screen to a player who is dribbling
9	Zone where the screen occurs	A0-D1) Various coded zones on the court (Figure 1)
10	Form	BACK) Back screen; FRONT) Front screen
11	Type of screen	DIAG) Diagonal screen; HOR) Horizontal screen; VERT) Vertical screen
12	Screen laterality	RIGHT) Right side; LEFT) Left side; CEN) Center
13	Type of combined defense on screen	SWT) With a switch; NSWT) No switch; DF2 × 1) 2-on-1 defense (Trap)
14	Type of defense on the ball handler's screen	d10) Go under (first level); d20) Go over (second level); d30) Hedge (third level); d40) Hard show (fourth level)
15	Action after ball screen (ball handler)	BOTFALT) Dribbler fouled; BOTPERD) Dribbler loses possession; PASBLOQ) Pass to screener; PASOTR) Pass to teammate; BOTTIR) Dribbler shoots; RPKFALT) Repick and fouled; RPKPERD) Repick and turnover; RPKPAS) Repick and pass; RPKTIR) Repick and shot; TIRBLOQFALT) Shot while screener commits a foul
16	Action After ball screen (screener)	NREC) Screener does not receive a pass; FALTBLOQ) Screener fouled during screen; BLOQTIR) Screener receives pass and shoots; BLOQFALT) Screener receives pass and is fouled; BLOQPERD) Screener receives pass and loses possession; BLOQPAS) Screener passes to a teammate.
17	Resolution of the screen	SHOT) Shot; CONTI) Continuity; FALT) Foul; PERD) Turnover; LUCH) Jump ball; FFB) Favor sideline out; CFB) Opponent sideline out; FFF) Favor baseline out; CFF) Opponent baseline out
18	Type of shot	T2) Two-point shot; T3) Three-point shot
19	Zone where action ends	NFIN) No finalization; A0-D1a) Various coded finishing zones (Figure 1)
20	Foul	FALT1) Foul committed; NFALT) No foul
21	Type of foul	FALTDF) Defensive foul; FALTATA) Offensive foul
22	Number of passes to finalize	P0) Zero passes; P1) One pass; P2) Two passes; P3) Three passes; M3) More than three passes
23	Success	BASK) Basket made; MNBASK) No basket; BASKFT) Basket and additional free throw; MNBASKFT) No basket but free throws

**Figure 1:**
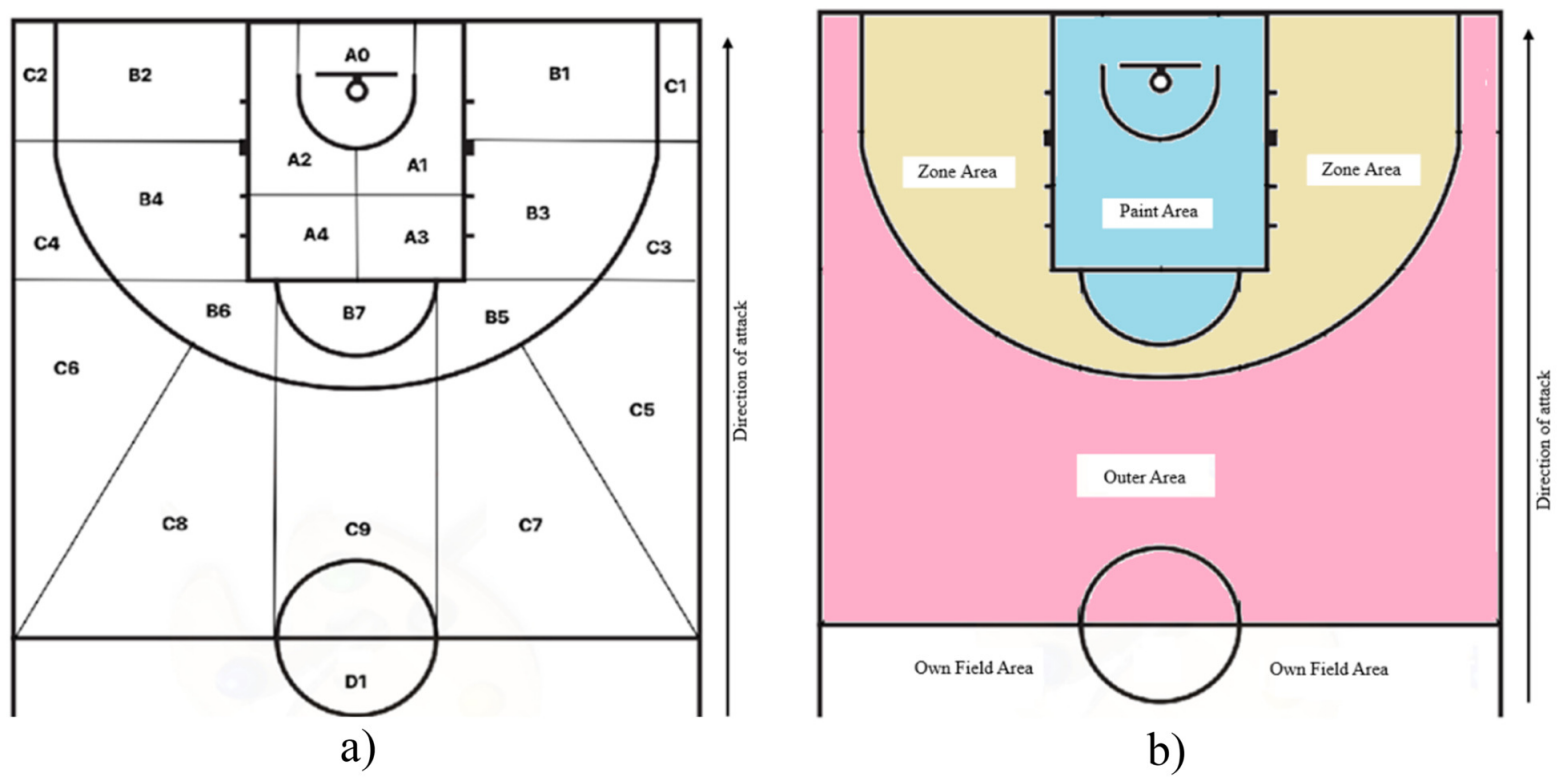
Court diagram. **(a)** Spatial distribution. **(b)** Area distribution.

To identify the zones where the pick and roll has been executed, a specific court diagram has been developed, which can be consulted in Figure 1.

### Procedure and recording quality

Two observers recorded the data for this research, both holding degrees in Physical Activity and Sport Sciences, with over 10 years of experience in the sport modality and expertise in observational methodology. Prior to the final coding process that would form the final data package for the research, following the recommendations established by Losada & Manolov (27) as well as those proposed by Anguera (28), observer training was conducted with the aim of minimizing coding errors.

To determine the reliability of the data obtained from the observation instrument, the measure of agreement for nominal classifications, where there is no ordering between categories, was used: Cohen's Kappa (29). This statistical coefficient is used to quantify the degree of agreement between observers, correcting for chance factors, mathematically expressed as: Kappa = (Po - Pe)/(1 - Pe), where “Po” is the observed percentage and “Pe” is the percentage expected by chance.

The entire sample was recorded by Observer 1, while Observer 2 recorded 15% of the total (30) to calculate the agreement coefficient, which yielded an overall value of 0.93, indicating “almost perfect” agreement according to the Landis & Koch scale (31). This analysis was performed using the Gseq v.5.1 software (32), ensuring the quality of the data.

Data recording and coding were carried out using the LINCE PLUS software (33), obtaining type IV data, concurrent and time-based (34).

### Data analysis

For data analysis, by searching for the associative relationship between categorical variables, Pearson's chi-square statistic (*χ*^2^) was used, applying the following formula: *χ*^2^ = ∑k i,j = 1 [(Fij – F^ij)^2^/F^ij]. This statistic allows determining the degree of association between the variables and the different criteria of success and construction of the offensive action to be analyzed. To examine the relationship between predictor variables and match outcomes, adjusted standardised residuals were calculated. These residuals allowed us to determine whether the observed frequencies differed significantly from the expected frequencies, which would indicate an association between the variables. Residuals were considered statistically significant if their absolute values exceeded 2.0, which corresponds to a 95% confidence interval. The magnitude of the association was assessed using Cramer's V statistic, which provides a measure of effect size. The interpretation of this statistic was done following Cohen's (35) criteria, which vary according to the degrees of freedom. The degrees of freedom for Cramer's V was calculated as the smaller value between (R-1) and (C-1), where R represents the number of rows and C the number of columns, following the methodology of Gravetter and Wallnau (36).

The implementation of the multivariate logistic regression technique allows us to statistically test the predictive model. Logistic regression is a statistical model used to estimate the probability of a categorical outcome (binary or multinomial) based on multiple independent variables. It applies the logistic (sigmoid) function to transform linear combinations of predictors into probabilities constrained between 0 and 1 (37). This method enables the simultaneous analysis of adjusted effects of predictors while controlling for confounding factors, making it a robust tool for predictive modeling.

The logistic regression procedure was carried out in two stages. First, a bivariate analysis was conducted using contingency tables and a chi-square test to assess whether there was a statistically significant relationship between success and the independent variables included in the observation instrument. Then, at the multivariate level, binary logistic regression was applied to test the prediction model.

Binary logistic regression is particularly useful for predicting the probability of a binary event (e.g., success/failure) based on multiple independent variables. This statistical technique is considered a supervised classification algorithm (machine learning), as it is used for predictive modeling in large datasets. It has been widely implemented in sports analytics, particularly in team sports, due to its ability to estimate the likelihood of different game-related events occurring.

A key characteristic of binary logistic regression is that the dependent variable must be dichotomous, meaning it has only two possible outcomes. Additionally, its applicability is supported by its high intrinsic explainability, as the model's coefficients allow for interpreting how each predictor increases or modifies the odds ratio in favor of a positive outcome.

For this study, the SPSS software (version 20.0) was used to perform the logistic regression analysis, ensuring statistical rigor in the estimation of probabilities and predictive power.

## Results

Of the 298 pick and roll plays analyzed during the different games that make up the study sample, 71.8% ended in a shot, compared to 28.2% that did not. Among the actions that ended in a shot, a distinction is made between those that directly result in a shot (SHOT) by one of the players directly involved in the action (the screener and the ball handler), and those in which, after the screen, the action continues with other shooting options through a player who is not directly involved in the screen action (Continuity). Additionally, the success rate of each of these actions was studied: Success (Basket -BASK- and Basket plus additional free throw -BASKFT-) and Failure (Missed Basket -MNBASK- and Missed Basket plus additional free throw -MNBASKFT-).

The results obtained from the study of pick and roll actions in each quarter of play in relation to the match played - Table 2- do not show significant differences (*χ*^2^ = 3.601; df = 9; *p* ≥ .939).

**Table 2:** Distribution of the pick and roll actions produced in each quarter of play in relation to the match played.

	Firstq	Secondq	Thirdq	Fourthq
SMF1	29,1%	21,5%	20,3%	29,1%
SMF2	22,4%	31,3%	23,9%	22,4%
3rd and 4th Place	24,1%	25,3%	22,9%	27,7%
Final	29,0%	24,6%	18,8%	27,5%

SMF1) Semifinal 1; SMF2) Semifinal 2; Firstq) First quarter; Secondq) Second quarter; Thirdq) Third quarter; Fourthq) Fourth quarter.

According to the results obtained from the analysis of offensive pick and roll actions that end in a shot and the game analyzed (Table 3), percentage differences are observed between the semifinal games (SMF1 and SMF2) and the final games (final -1st and 2nd place- and consolation final -3rd and 4th place-), with higher percentages of actions finishing in a shot in the semifinals (81% and 76.1%) than in the finals (67.5% and 62.3%). Although the results from the Chi-square test were not significant, they are close to significance (*χ*^2^ = 7.763; df = 3; *p* = .051).

**Table 3:** Relationship between the games played and the completion of the action with a shot.

	Does not end the action with a shot	Ends the action with a shot
SMF1	19.0%	81.0%
SMF2	23.9%	76.1%
3rd and 4th Place	32.5%	67.5%
Final	37.7%	62.3%

SMF1) Semifinal 1; SMF2) Semifinal 2.

In Table 4, the results obtained from the analysis of the different games played and the resolution of the screen, for those actions that end in a shot, are presented. In most games, the direct screen action is resolved with a shot by one of the involved players (SHOT) at high rates (68.8% in SMF1, 74.5% in SMF2, and 67.4% in the Final). However, in the third-place game, the most frequent resolution is CONTI (play continuation by a non-screened player), with 50.9% of actions ending in a shot.

**Table 4:** Relationship of actions that end in a shot, performed through continuity (CONTI) or among the players involved in the screen (SHOT), by game studied.

		SHOT	CONTI
SMF1	Frequency	44	20
	Percentage	68,8%	31,3%
	ASR	,8	-,8
SMF2	Frequency	38	13
	Percentage	74,5%	25,5%
	ASR	1,7	−1,7
3rd and 4th Place	Frequency	27	28
	Percentage	49,1%	50,9%
	ASR	−2,8	2,8
Final	Frequency	29	14
	Percentage	67,4%	32,6%
	ASR	,4	-,4

SMF1) Semifinal 1; SMF2) Semifinal 2; ASR) adjusted standardised residuals SHOT) Shot; CONTI) Continuity.

The results corresponding to the Chi-square test show significant differences between them, specifically in the actions that end through the development of play continuity in the third and fourth place game (*χ*^2^ = 8.627; df = 3; *p* = .035; Cramér's V = .201) – [number of cases = 213].

Furthermore, significant differences (*χ*^2^ = 16.430; df = 1; *p* ≤ .001) are found between the value of 2-point shots and 3-point shots based on the resolution of the screen. Table 5 presents the results from the analysis between the resolution of the screen and the value of the shot taken. It can be observed that for plays where the resolution of the screen is SHOT, 2-point shots account for 81.9%, while 3-point shots make up 18.1%. In contrast, when the resolution is CONTI (continuity), 56% of the shots are 2-point shots, and 44% are 3-point shots.

**Table 5:** Relationship between actions resulting from the direct screen (shot and conti) and the value of the shots executed (2 point shot and 3 points shot).

	2-point shot	3-point shot
SHOT	81.9%	18.1%
CONTI	56.0%	44.0%

SHOT) Shot; CONTI) Continuity.

The results corresponding to the study of the success of the shots (hit or miss) and the value of the shots executed after the resolution of the screen by one of the two players directly involved in its execution (SHOT), show that 2-point shots have the highest success rate (43.4%), compared to 28% for 3-point shots Table 6. The results obtained from the chi-square test do not show significant differences (*χ*^2^ = 2.004; df = 1; *p* = .157).

**Table 6:** Relationship of success (hit or miss) of 2-point and 3-point direct shots (executed by one of the two players involved in the direct screen action).

	Hit	Miss	Total shots
2-Point shot	43.4%	56.6%	81,9%
3-Point shot	28.0%	72.0%	18,1%

Based on the analysis of the success of the shots (hit or miss) and the value of the shots executed after the resolution of the screen by a player who does not directly participate in the execution of the direct screen (CONTI), the results obtained – Table 7 show that 2-point shots have the highest success rate (42.9%) compared to 30.3% for 3-point shots. No significant differences were found (*χ*^2^ = 1.245; df = 1; *p* = .265) in the chi-square test analysis.

**Table 7:** Relationship of success (hit or miss) of 2-point and 3-point shots in continuity (executed by a player not involved in the direct screen action).

	Hit	Miss	Total shots
2-Point Shot	42.9%	57.1%	56%
3-Point Shot	30.3%	69.7%	44%

After studying the type of shot, an analysis was conducted on the number of passes made and the outcome of the shot (hit or miss), considering whether the shot was executed by one of the players directly involved in the screen (SHOT) or by another player (CONTI). In Table 8, it can be seen that, among those plays finalized by one of the players directly involved in the direct screen, the highest percentage of success is achieved by those that involve a pass, with 56.8%, while those that are finished without a pass (i.e., when the ball handler completes the action) reach 33% success. On the other hand, in plays that are finished through at least one player who does not directly participate in the direct screen (CONTI), 42.9% of the plays end in a basket after making one pass following the screen, 38.1% after two passes, and 9.1% after three passes following the screen.

**Table 8:** Relationship between the number of passes executed before the shot and the success (hit or miss) achieved, for SHOT and CONTI actions.

No. of passes	Hit	Miss
SHOT		
0 Passes	33.0%	67.0%
1 Pass	56.8%	43.2%
CONTI		
1 Pass	42.9%	57.1%
2 Passes	38.1%	61.9%
3 Passes	9.1%	90.9%

SHOT) Shot; CONTI) Continuity.

Based on the results obtained from the Chi-square tests, significant differences were found in the completion of the basket (hit) based on the number of passes executed (*χ*^2^ = 7.064; df = 1; *p* = 0.008) in plays that are directly finished by one of the players involved in the screen (TIR). However, no significant differences (*p* ≥ 0.187) were observed in plays where the resolution of the screen is continuity (CONTI).

Finally, we analysed the type of action performed by the ball holder and the result of the shot (hit or miss). Table 9 shows that the plays finished by a direct individual action of the ball possessor have a success rate of 25.2%, while collective actions have a higher success rate - 32.10% - and no significant differences were found (x^2^ = 1.617; df = 1; *p* = 0.203) in this respect. On the other hand, when analysing collective play - Table 10, the pass to the defender has the highest success rate, with 41.7%, compared to 25% success rate for passes to a third player. In this case, significant differences can be seen between the variables studied (x^2^ = 5.240; df = 1; *p* = 0.022).

**Table 9:** Relationship between the action taken by the ball holder before the shot (individual action and pass to a teammate) and the success (hit or miss) obtained.

Action	Hit	Miss
Individual action	25,20%	74,80%
Pass to a teammate	32,10%	67,90%

**Table 10:** Relationship between the action performed by the teammate receiving the pass (pass to the screener and pass to a third player) and the success (hit or miss) obtained.

Action	Hit	Miss
Pass to the screener	41.7%	58.3%
Pass to a third player	25.0%	75.0%

### Binary logistic regression results

Next, to understand the effectiveness of the model, the variable success was reconfigured, establishing it as dichotomous. To do this, the variables BASK, BASKFT were set as success, and the variables MNBASK and MNBASKFT as non-success.

For the final model, four variables showed the largest increase in the odds ratio in favor of Completion of action Table 11. In this case, although the overall predictive ability of the model is 0.274, we find significant results in other categories such as the second quarter (*p* = 0.024, Exp(B) = 2.391) or third quarter (*p* = 0.034, Exp(B) = 2.366). In this case, the odds ratio indicates an almost 2.5-fold increase in the probability of success for both quarters of the match. These categories have a strong impact, as their *p*-value indicates statistical significance and their Exp(B) are greater than 1, suggesting that they increase the probability of success. On the other hand, the category Type of Defence on the Ball Handler's Screen (d30) (*p* = 0.014, Exp(B) = 3.990) is also highly relevant, as its odds ratio indicates that it increases the probability of success by almost four times. In short, although the constant in this model is not a strong predictor, the factors that influence the prediction of success are Secondq, Thirdq and Type of Defence on the Ball Handler's Screen (d30), which have a much greater impact on the model. In addition, the independent variable “Hard show (fourth level)” increased the odds ratio for the “Completion of action” category by 3.990.

**Table 11:** Results of the binary logistic regression model.

	B	SE	Wald	df	p.	Exp(B)
Quarter (Firstq)			6,808	3	,078	
Quarter (Secondq)	,872	,387	5,077	1	,024	2,391
Quarter (Thirdq)	,861	,407	4,489	1	,034	2,366
Quarter (Fourthq)	,456	,364	1,566	1	,211	1,577
Type of defense on the ball handler's screen (d10)			6,610	3	,085	
Type of defense on the ball handler's screen (d20)	1,264	,692	3,339	1	,068	3,538
Type of defense on the ball handler's screen (d30)	1,384	,566	5,983	1	,014	3,990
Type of defense on the ball handler's screen (d40)	,996	,580	2,943	1	,086	2,707
Constant	-,638	,583	1,195	1	,274	,528

B) logistic regression coefficient; SE) standar error; Firstq) First quarter; Secondq) Second quarter; Thirdq) Third quarter; Fourthq) Fourth quarter; d10) Go under (first level); d20) Go over (second level); d30) Hedge (third level); d40) Hard show (fourth level).

The model has a specificity of 44.0% and a sensitivity of 98.6%. The overall classification accuracy was 72.6%. The model is well-fitted according to the result of Nagelkerke's R^2^ = 0.69.

## Discussion

The goal of this study was to analyze the effectiveness of the pick-and-roll in top-level women's basketball and describe its most effective completion methods. The results confirm that the distribution of the pick and roll during a basketball game tends to remain relatively uniform throughout the four quarters, aligning with previous findings reported by Nunes et al. (7). However, a decrease in the frequency of this play was observed during the finals, which could be explained by teams opting for more conservative strategies in high-pressure contexts, where safe plays are prioritized to minimize errors. This observation is consistent with studies suggesting that, under critical situations, teams tend to adjust their playing style to ensure more effective shots (8).

Regarding the success of pick-and-roll plays, it was identified that passes directed to the player running the screen significantly increase the probability of scoring a basket, compared to individual action or the participation of a third player outside the pick-and-roll, as they tend to be in a position closer to the basket, favouring a higher shooting percentage on two-point attempts. This finding aligns with the conclusions of Bardavío et al. (8), who highlighted that the effectiveness of this play is closely related to the players' ability to identify and exploit defensive advantages through adequate spatial and temporal coordination. Similarly, Suárez-Cadenas et al. (6) highlighted that passes to players in optimal positions close to the basket tend to result in more efficient finishes.

Moreover, the off-ball movements of players play a critical role in creating space that favors the success of outside shots. In this regard, the results are consistent with studies that emphasize the importance of game fluidity and the active involvement of players not directly participating in the screen, as their ability to get open increases the likelihood of success in three-point shots (38). This finding underscores the need to integrate strategies that consider the collective movement of players to maximize the effectiveness of perimeter shots, which, combined with interior movements, improve the passer's decision-making (39).

Furthermore, it was found that pick-and-roll plays resolved with a single pass have a higher probability of success. These findings may be due to limited defensive reaction time (40). In contrast, when multiple passes are executed, defensive reorganization reduces the chances of effective completion, as highlighted by previous studies on the relationship between execution speed and success in offensive plays (6, 41). The results regarding the pass receiver, specifically the player performing the screen (41,7% of success), also align with findings from Nunes et al. (7), who argue that a quick pass to the player executing the screen—who is generally in a better position to finish near the basket—is a more efficient strategy than distributing the ball to other players with a lower success probability.

Overall, the findings from this study highlight the importance of coordination, speed, and precision in executing the pick-and-roll to maintain its effectiveness, especially in critical moments like finals, where strategies are often more conservative. These results reinforce the idea that reading the game and adapting to competitive circumstances are key elements in the success of this play. However, further exploration is necessary to understand how game dynamics and situational pressure affect the implementation and effectiveness of the pick-and-roll, as these factors may vary depending on the level of competition and the specific characteristics of the teams.

Regarding the multivariate results, the model has a significant explanatory capacity (69%). The variables included value the second and third quarters as the decisive ones where the pick & roll is used. These results present a clear difference with respect to men's basketball, where there is still no scientific consensus, in view of the available studies (10, 42, 43). However, what does seem clear is that, both in the men's and women's categories, the importance of the pick & roll to create numerical superiority or optimal attack spaces is an essential resource for teams (3), being a fundamental tool in the offensive process.

A key strength of this study is its ecological validity, which enhances the applicability of the present findings to real-world settings (44) However, some limitations should be acknowledged, including the limitation of working with a sample that is limited to the analysis of a specific championship, which may compromise the representativeness of the findings. A small number of participants in a specific event may not adequately reflect the heterogeneity of the elite women's basketball population in terms of playing styles, skill levels or strategies. Therefore, observations made in such a limited context should be interpreted with caution, recognising their potential specificity and the difficulty in extrapolating them to the general dynamics of elite women's basketball. In this sense, increasing the sample of selected matches as well as the number and type of championships could help to generalise the results and help in the advancement of this sport.

Likewise, the present study presents the limitation of a single test to confirm the validity of the data recorded, the inter-observer concordance which, although sufficient to determine the reliability of the data, can be complemented with other tests, such as intra-observer concordance or the application of the theory of generalisability -TG- (45). These aspects would provide greater robustness to the reliability of the data recorded, as well as the possibility of generalising the results obtained, providing a solution closer to the aforementioned limitation.

## Conclusion

The analysis conducted allows for the identification of direct screen plays as generating various finishing opportunities that vary depending on the number of passes made and the players involved in the action. Specifically, it was observed that these opportunities include immediate shots taken by the player benefiting from the screen or by the screener, as well as the involvement of other players not directly engaged in the action, generally after one to three passes.

The highest effectiveness of the pick-and-roll occurs when the player executing the screen finishes the action by receiving the pass and taking a shot near the basket. These high-proximity areas, offering more favorable angles and lower levels of defensive obstruction, maximize the likelihood of scoring. This finding emphasizes the importance of prioritizing actions that facilitate finishes close to the basket to optimize offensive outcomes.

In situations where the player benefiting from the screen cannot execute an effective shot, the most frequently chosen solution is to finish the play with an outside shot. This decision reflects the team's ability to adapt to defensive circumstances, employing space creation and shooting accuracy from a distance as an alternative resource.

Additionally, the results demonstrate that the effectiveness of the screen diminishes as the temporal or spatial distance between the screen execution and the final shot increases. In other words, the closer the relationship between the screen and the finish, the higher the likelihood of scoring. This effect suggests that the influence of the screen weakens as the action prolongs, reinforcing the need to capitalize on the immediate advantages generated by this play.

Finally, the most effective zones for scoring after a screen are those near the basket or in intermediate areas within the key, where shots typically occur with minimal defensive obstruction. This finding highlights the importance of efficient occupation of advantageous spaces, allowing players to optimize the opportunities created by the screen, both in terms of accuracy and accessibility for the shot.

As regards the multivariate analysis, the available results highlight the second and third quarters as the most important for the team's offensive success.

These conclusions provide new perspectives on the tactical impact of direct screens in basketball and suggest strategic approaches focused on maximizing proximity and fluidity in plays to increase offensive effectiveness.

## Data Availability

The raw data supporting the conclusions of this article will be made available by the authors, without undue reservation.
